# Identification of RNA Methylation-Related lncRNAs Signature for Predicting Hot and Cold Tumors and Prognosis in Colon Cancer

**DOI:** 10.3389/fgene.2022.870945

**Published:** 2022-04-06

**Authors:** Rong He, Changfeng Man, Jiabin Huang, Lian He, Xiaoyan Wang, Yakun Lang, Yu Fan

**Affiliations:** ^1^ Cancer Institute, The Affiliated People’s Hospital of Jiangsu University, Zhenjiang, China; ^2^ Department of Gastroenterology, The Affiliated Suqian First People’s Hospital of Nanjing Medical University, Suqian, China

**Keywords:** colon cancer, RNA methylation, long non-coding RNA, immunotherapy, tumor immune microenvironment

## Abstract

N6-methyladenosine (m6A), N1-methyladenosine (m1A), 5-methylcytosine (m5C), and 7-methylguanosine (m7G) are the major forms of RNA methylation modifications, which are closely associated with the development of many tumors. However, the prognostic value of RNA methylation-related long non-coding RNAs (lncRNAs) in colon cancer (CC) has not been defined. This study summarised 50 m6A/m1A/m5C/m7G-related genes and downloaded 41 normal and 471 CC tumor samples with RNA-seq data and clinicopathological information from The Cancer Genome Atlas (TCGA) database. A total of 1057 RNA methylation-related lncRNAs (RMlncRNAs) were identified with Pearson correlation analysis. Twenty-three RMlncRNAs with prognostic values were screened using univariate Cox regression analysis. By consensus clustering analysis, CC patients were classified into two molecular subtypes (Cluster 1 and Cluster 2) with different clinical outcomes and immune microenvironmental infiltration characteristics. Cluster 2 was considered to be the “hot tumor” with a better prognosis, while cluster 1 was regarded as the “cold tumor” with a poorer prognosis. Subsequently, we constructed a seven-lncRNA prognostic signature using the least absolute shrinkage and selection operator (LASSO) Cox regression. In combination with other clinical traits, we found that the RNA methylation-related lncRNA prognostic signature (called the “RMlnc-score”) was an independent prognostic factor for patients with colon cancer. In addition, immune infiltration, immunotherapy response analysis, and half-maximum inhibitory concentration (IC50) showed that the low RMlnc-score group was more sensitive to immunotherapy, while the high RMlnc-score group was sensitive to more chemotherapeutic agents. In summary, the RMlnc-score we developed could be used to predict the prognosis, immunotherapy response, and drug sensitivity of CC patients, guiding more accurate, and personalized treatment regimens.

## Introduction

Colon cancer (CC), a common gastrointestinal malignancy, is the third leading cause of cancer-related mortality, and morbidity worldwide (Siegel et al., 2022). Although patient prognosis has significantly improved with the advances in surgery, radiotherapy, and chemotherapy techniques, the 5-years survival rate for patients with advanced CC is only 10% (Su and Zhang, 2017). In recent years, immunotherapy has shown excellent anti-tumor efficacy in many types of malignancies, such as colon cancer, head and neck tumors, melanoma, kidney cancer, and lung cancer (Constantinidou et al., 2019; Morse et al., 2020). However, not all CC patients respond to immunotherapy. Patients who benefit from immunotherapy are mainly those with mismatch repair-deficient (dMMR) or microsatellite instability-high (MSI-H), with an efficacy rate of only 30–40%, and this population represents only a small fraction of those with advanced CC(Le et al., 2017; Morse et al., 2020). Other immunotherapeutic biomarkers include tumor mutational burden (TMB) and programmed cell death ligand-1 (PD-L1) expression (Chan et al., 2019; Luchini et al., 2019; Sagredou et al., 2021). However, the above markers have significant limitations in clinical application, and there exist some patients who are negative for the above markers and can also benefit from PD-1/PD-L1 based immunotherapy (Liu et al., 2019; He et al., 2021). Therefore, it is urgent to find some novel and effective biomarkers to detect the prognosis of CC and to guide immunotherapy regimens.

RNA methylation is considered an important process in epigenetic regulation, which occurs in mRNA and in ncRNA (Xu et al., 2021). Various forms of RNA methylation exist depending on the site of methylation, including N1-methyladenosine (m1A), 5-methylcytosine (m5C), N6-methyladenosine (m6A), 7-methylguanosine (m7G), and 2-O-dimethyladenosine (m6Am) (Xie et al., 2020). RNA methylation is involved in various physiological and pathological processes, and its dysregulation is closely associated with the development of human cancer. For example, the m6A-related regulator METTL3 was found to be highly expressed in several types of cancers and associated with poor prognosis, including gastric cancer (Wang et al., 2020), liver cancer (Chen et al., 2018), and colon cancer (Li et al., 2019a). The m5C-related factors form a tumor microenvironment suitable for migration and metastasis of various cancer cells by regulating some known tumor promoters, such as HDGF, TGF-β, FGF2, and G3BP1(Zhang et al., 2021c). The m1A demethylase ALKBH3, also known as prostate cancer antigen 1 (PCA-1), in addition to being exceptionally abundant in prostate cancer (Konishi et al., 2005), the oncogenic role of m1A demethylation has been found in colon (Zhao et al., 2019), breast (Woo and Chambers, 2019), and lung cancers (Tasaki et al., 2011). METTL1/WDR4-mediated enhancement of m7G modification improves translation efficiency and is associated with poor prognosis in several cancers (Katsara and Schneider, 2021). In addition, recent studies have demonstrated that RNA methylation can play a critical role in tumor immunity by affecting immune cell maturation and RNA immunogenicity, which provides a new direction for future cancer immunotherapy (Zhang et al., 2021a).

Long non-coding RNAs (lncRNAs) are a class of non-protein-coding RNAs with transcripts longer than 200 nt, mainly involved in epigenetic regulation, transcriptional, and post-transcriptional regulation (Cao et al., 2019). Increasing evidence suggests that lncRNAs play an integral role in the development and progression of several cancers, including colon cancer, suggesting that they could serve as novel biomarkers, and therapeutic targets (Meng et al., 2021; Dong et al., 2022; Shen et al., 2022). In recent years, studies on the relationship between RNA methylation and lncRNA in tumors have become the hot topic. For example, NSUN2-mediated m5C methylation of lncRNA H19 may contribute to the development and growth of hepatocellular carcinoma by affecting the interaction with oncoprotein G3BP1 (Sun et al., 2020). ALKBH5 promotes the invasion and metastasis of gastric cancer cells by demethylating lncRNA NEAT1 (Zhang et al., 2019). Wang et al. developed an m5C-related lncRNA prognostic model to predict patient prognosis (Wang et al., 2021b). Zhang et al. constructed a risk model including 31 m6A-related lncRNAs in colon cancer that could be used to predict patient prognosis (Zhang et al., 2021b). However, studies including four major (m6A, m1A, m5C, and m7G) RNA methylation modification-related lncRNAs in tumors have remained relatively rare so far. In this study, we collected transcriptomic data and clinical information from CC patients and performed a series of bioinformatic analyses to understand the expression of m6A, m1A, m5C, and m7G-RNA methylation modification-related lncRNAs and their impact in CC, and to elucidate the potential mechanisms of prognosis. The significance and originality of this study is that it further reveals a potential link between RNA methylation modification patterns and tumor microenvironment and clinical treatment response. This novel signature can be used to assess the sensitivity of CC patients to immunotherapy and chemotherapy.

## Materials and Methods

### Data Acquisition and Processing

Transcriptome profiling data, somatic mutation data, and corresponding clinical data for the TCGA-CORD cohort were downloaded from The Cancer Genome Atlas (TCGA) database (https://cancergenome.nih.gov/), including data from 471 CC and 41 normal case samples. Gene expression profiles were then fully annotated with the Gencode project (Frankish et al., 2019) and distinguished into mRNAs and lncRNAs profiles. The GSE17536 dataset (N = 177) was obtained from Gene Expression Omnibus (GEO, http://www.ncbi.nlm.nih.gov/geo) as an external validation set to better verify the role of target lncRNAs.

### Differential Expression and Mutational Analysis of RNA Methylation Regulators

Through the review of the latest literature, a total of 50 m6A-, m1A-, m5C-, and m7G-RNA methylation regulators were obtained. Among them, 25 m6A regulators (METTL3, METTL14, METTL16, WTAP, KIAA1429, VIRMA, RBM1, RBM15, RBM15B, and ZC3H13, FTO, ALKBH5, YTHDC1, YTHDC2, YTHDF1, YTHDF2, YTHDF3 IGF2BP1, IGF2BP2, IGF2BP3, HNRNPA2B1, HNRNPC, HNRNPG, RBMX, LRPPRC, and FMR1) (Li et al., 2019b; Hu et al., 2019; An and Duan, 2022), 13 m1A regulators (TRMT6, TRMT61A, TRMT61B, TRMT61C, TRMT10C, BMT2 RRP8, YTHDF1, YTHDF2, YTHDF3, and YTHDC1, ALKBH1, and ALKBH3) (Xie et al., 2020; Song et al., 2021a), 14 m5C regulators (NOP2, NSUN1, NSUN2, NSUN3, NSUN4, NSUN5, NSUN7, DNMT1, TRDMT1, DNMT3A, DNMT3B, TET2, YBX1, and ALYREF) (Meng et al., 2021), and 2 m7G regulators (METTL1 and WDR4) (Tomikawa, 2018) were included. RNA methylation regulators differentially expressed in colon cancer and normal tissues in the TCGA-CORD cohort were identified using the “limma” package. The “maftools” package was used to generate mutation maps of RNA methylation regulators in CC patients. CNV altered positions of RNA methylation regulators on 23 chromosomes were mapped using the “RCircos” package.

### Identification of RNA Methylation-Related lncRNA and Analysis of Their Prognostic Value

Pearson correlation analysis was used to screen for lncRNAs co-expressed with differentially expressed RNA methylation-related genes (|Pearson R|>0.5 and *p*-value <0.001). Univariate Cox regression analysis was performed to screen for RMlncRNAs significantly associated with OS (*p* < 0.05), and the Sankey diagram was mapped by the “ggalluvial” R package. The Wilcoxon test was used to detect differences in the expression of prognosis-related RMlncRNAs between tumor tissues and normal tissues.

### Consistent Clustering of RNA Methylation-Related lncRNAs

Based on the expression of RMlncRNAs with prognostic value, unsupervised consensus clustering was performed using “ConsensusClusterPlus” on 433 colon cancer patients to identify potential molecular subtypes (Wilkerson and Hayes, 2010). R packages “ survival” and “survminer” were used to analyze the prognosis of samples with different molecular subtypes. Clinical data were included and analyzed for differences in molecular subtypes by using the “heatmap” R package for distinct clinicopathological features. The proportion of 22 tumor-infiltrating immune cells (TICs) in each sample was quantified using the CIBERSORT algorithm (Newman et al., 2015). The ESTIMATE algorithm was used to calculate the tumor microenvironment (TME) score (including immune score, stromal score, ESTIMATE score, and tumor purity) for each sample (Yoshihara et al., 2013). In addition, we synthesized 38 immune checkpoint genes from the literature and examined the expression of these checkpoint genes among molecular subtypes (Pardoll, 2012; Nirschl and Drake, 2013).

### Construction and Validation of RNA Methylation-Related lncRNA Signature

The TCGA-CORD cohort was randomly divided into a training set and a test set (1:1 ratio). A minimum absolute shrinkage and selection operator (LASSO) Cox regression analysis was used to narrow down candidate lncRNAs and develop an RNA methylation-related lncRNA signature (we named it RMlnc-score). The formula is as follows: RMlnc-score = Σ (βi × Expi) (β: coefficients, Exp: lncRNA expression level). Patients were then divided into high RMlnc-score and low RMlnc-score groups based on the median value of RMlnc-score. Kaplan-Meier survival curves were plotted using the R package “survival” to describe the overall survival difference between the high and low score groups. Receiver operating characteristic curves (ROC) analysis was performed to evaluate its sensitivity and accuracy. Heatmaps were generated to reveal differences in signature lncRNA expression in the low and high RMlnc-score groups.

### Analysis of the Prognostic Value and Clinical Relevance for the RMlnc-Score

The student’s t-test was used to assess the relationship between RMlnc-score and clinical characteristics. In addition, survival analysis was performed to further elucidate the relationship between RMlnc-score by sex (male and female), age (≤65 and >65 years), T-stage (T1-2 and T3-4), N-stage (N0 and N1-2), M-stage (M0 and M1), and grade (stages I-II and stages III-IV) in each subgroup for prognostic ability. Subsequently, univariate and multivariate Cox regression analyses were used to determine the relationship and independence between clinicopathological characteristics and RMlnc-score. A nomogram and calibration curves were then constructed based on independent prognostic factors from multivariate Cox regression analysis to predict the probability of survival at 1, 3, and 5 years in CC patients. The GSE17536 dataset was used as an external validation cohort to further assess the prognostic value and clinical relevance of model lncRNAs.

### Principal Component Analysis and Assessment of Immune Cell Infiltration

The R package “scatterplot3d” was used to perform PCA analysis to explore potential differences between high and low RMlnc-score groups. To analyze the correlation between RMlnc-score and TICs, we used different software (including ssGSEA, xCELL, Timer, Quantiseq, MCPcounter, EPIC, CIBERSORT-ABS, and CIBERSORT) to comprehensively analyze of immune cell infiltration.

### Assessment of Response to Anti-Tumor Therapy

The tumor immune dysfunction and exclusion (TIDE) algorithm (Fu et al., 2020) was used to assess the potential response of colon cancer patients in the different RMlnc-score groups to immunotherapy. Data from the Genomics of Drug Sensitivity in Cancer (GDSC) database were used to predict the response of CC patients to chemotherapeutic drug therapy. The “pRRophetic” R package (Geeleher et al., 2014) was used to calculate the half-maximal inhibitory concentration (IC50) of common chemotherapeutic agents.

### Prediction of RNA Methylation Modification Sites on 7 lncRNAs

m6A-Atlas (Tang et al., 2021) and SRAMP(Zhou et al., 2016) were used to predict the m6A site of the lncRNAs; m5C-Atlas (Ma et al., 2022) and RNAm5Cfinder (Li et al., 2018) were used to predict the m5C site of the lncRNAs; m7GHub (Song et al., 2020) and iRNA-m7G (Chen et al., 2019) databases were used to predict the m7G site of the lncRNAs.

### Statistical Analysis

All statistical analyses were performed using R software (v4.0.2). *p* values < 0.05 were considered statistically significant if not explicitly stated.

## Results

### Landscape of RNA Methylation Regulator Expression and Gene Mutation in CC

**FIGURE 1:**
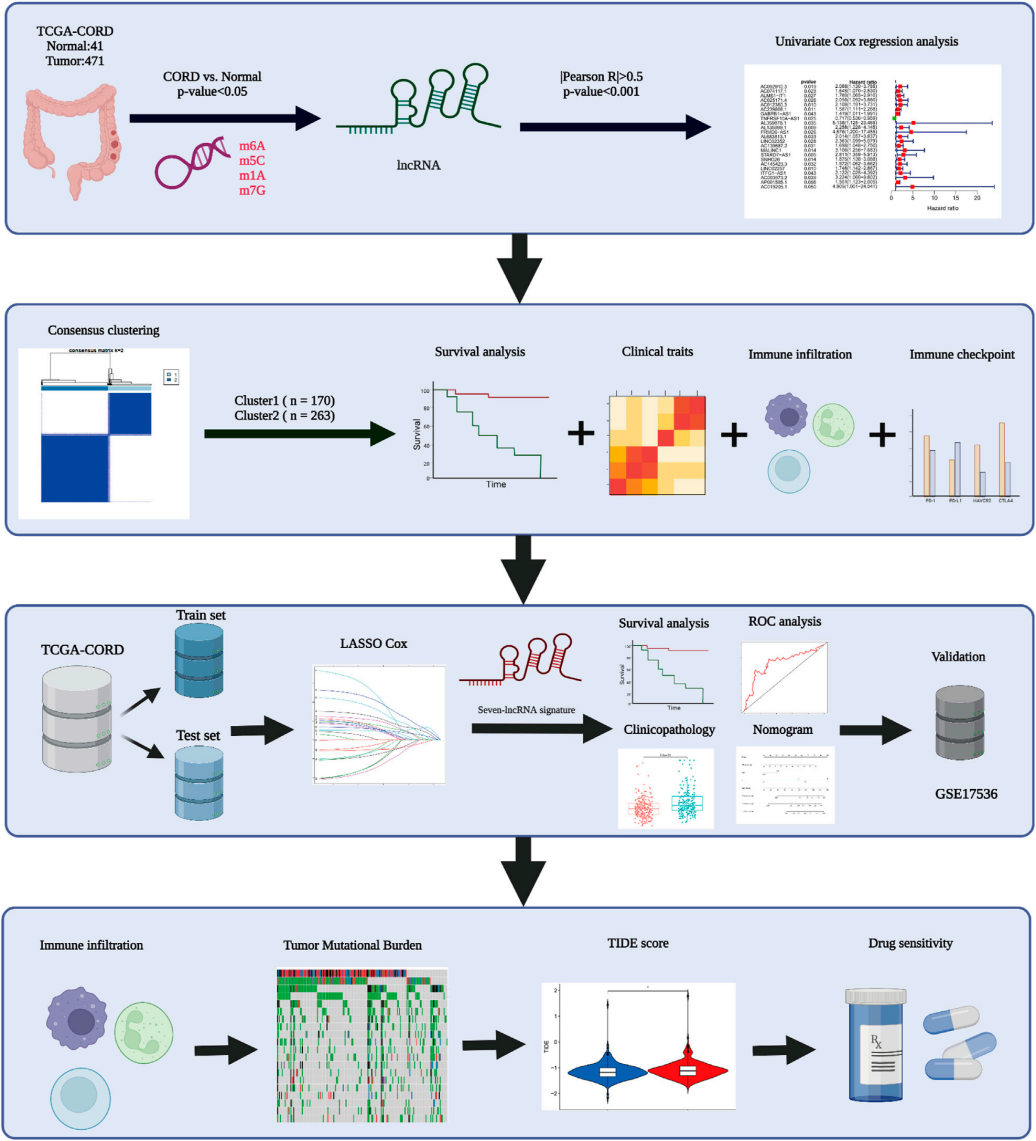
Workflow diagram of this study.

**FIGURE 2:**
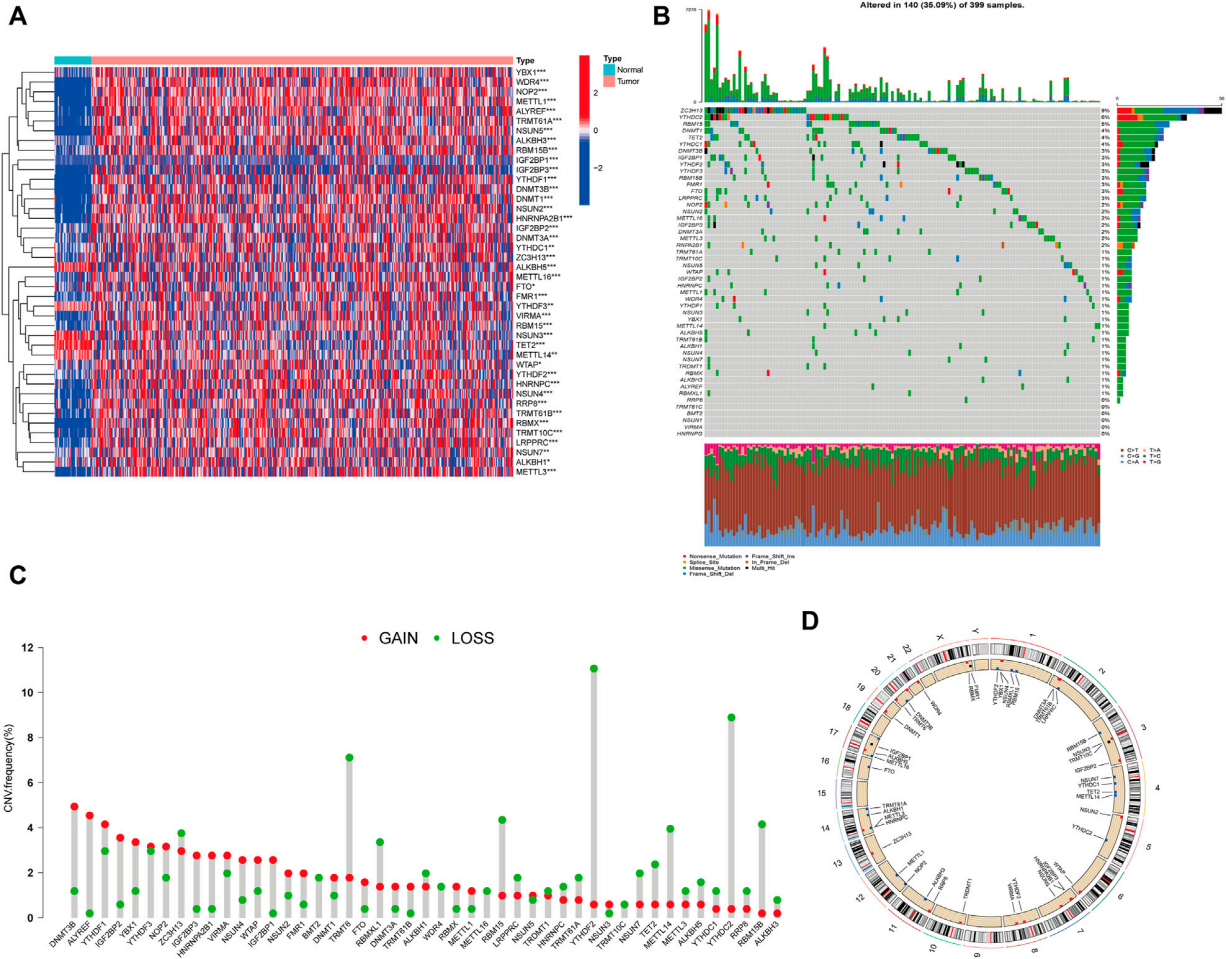
Characteristics and differences of RNA methylation-related regulators in CC. **(A)** Heatmap of differential expression of RNA methylation-related regulators between normal (*n* = 41) and colon cancer tissues (*n* = 471) in the TCGA-CORD cohort. **(B)** Mutation waterfall plots of 399 colon cancer patients from the TCGA-CORD cohort. **(C)** Copy number variation (CNV) frequency of RNA methylation-related regulators in the TCGA-CORD cohort. **(D)** The location of CNV alterations of RNA methylation-associated regulators on chromosomes in the TCGA-CORD cohort. *p<0.05; **p<0.01; ***p<0.001.

The workflow of this study is illustrated in Figure 1. First, we investigated the expression of 50 m1A-, m5C-, m6A-, and m7G-RNA methylation regulatory genes in the TCGA-CORD cohort (Figure 2A). The results showed that there were 42 differentially expressed RNA methylation regulatory genes. Among them, 37 regulators were highly expressed in colon cancer tissues, and five were lowly expressed in colon cancer tissues. Next, we investigated the incidence of somatic mutations and copy number variations for 50 regulators in TCGA-CORD. A total of 140 of 399 samples (35.09%) experienced genetic alterations in RNA methylation regulators (Figure 2B). Among them, ZC3H13 (9%) was the gene with the highest mutation frequency, followed by YTHDC2 (6%), and RBM15 (5%.) The investigation of CNV alteration frequency revealed that all RNA methylation regulators were found to show prevalent CNV alterations. Among them, DNMT3B, ALYREF, YTHDF1/3, IGF2BP2/3, YBX1, and HNRNPA2B1 showed significant copy number amplification, while TRMT6, YTHDF2, YTHDC2, and RBM15/15B showed remarkable copy number deletions (Figure 2C). Figure 2D shows the location of CNV changes in RNA methylation regulators on chromosomes. The above analysis revealed a high degree of heterogeneity in the expression and inherited variation status of RNA methylation in CC, demonstrating that RNA methylation-related regulators may play a pivotal position in the occurrence and development of CC.

### Identification of RNA Methylation-Related lncRNAs in CC Patients

**FIGURE 3:**
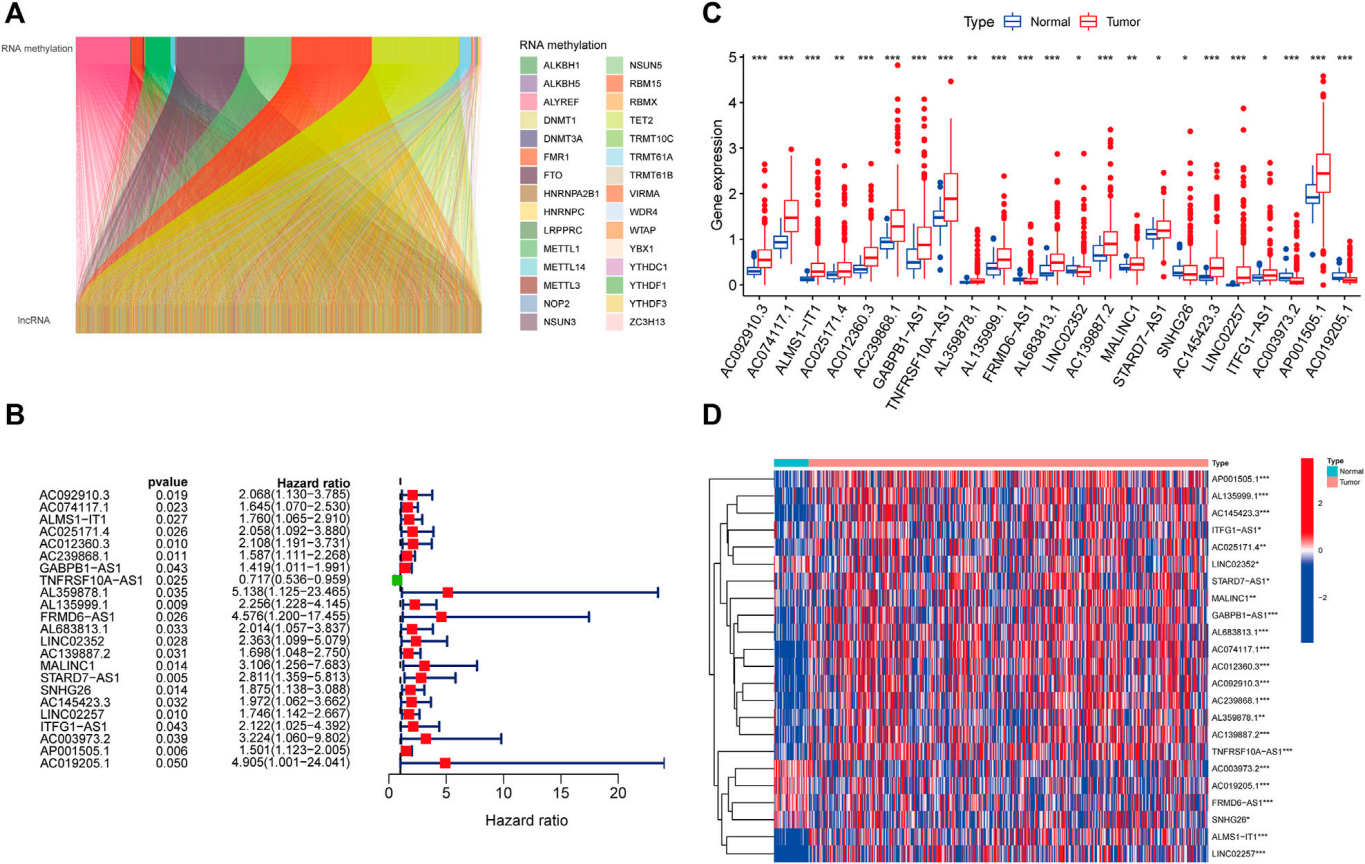
Identification of prognostic value of RNA methylation-related lncRNAs. **(A)** 1,057 lncRNAs were co-expressed with differentially expressed RNA methylation-related regulators. **(B)** Univariate Cox regression analysis screened 23 lncRNAs with prognostic value. **(C,D)** The boxplot and heatmap of 23 lncRNAs with prognostic value differentially expressed between 41 normal and 471 tumor tissues in the TCGA-CORD cohort. *p<0.05; **p<0.01; ***p<0.001; ns, no sense.

We identified 1,057 lncRNAs significantly associated with 42 differentially expressed RNA methylation regulators by using Pearson correlation analysis and defined them as RMlncRNAs. Based on the mRNA-lncRNA co-expression pattern, we constructed a Sankey diagram to show their linkage (Figure 3A). After excluding normal tissues or patients lacking survival data, we merged survival information with RMlncRNA expression data of colon cancer patients (final number of patients = 433). Subsequently, we performed univariate Cox regression analysis and found that 23 RMlncRNAs were significantly associated with OS of colon cancer patients (*p* < 0.05, Figure 3B). Among them, only TNFRSF10A-AS1 was identified as a protective factor with a risk ratio (HR) < 1, while all others were considered as risk factors. The bar graph and heatmap showed significant differences in the expression of these 23 prognosis-related RMlncRNAs between normal and colon cancer tissues (Figures 3C, D).

### Molecular Subtypes Mediated by 23 Prognosis-Related RMlncRNAs

**FIGURE 4:**
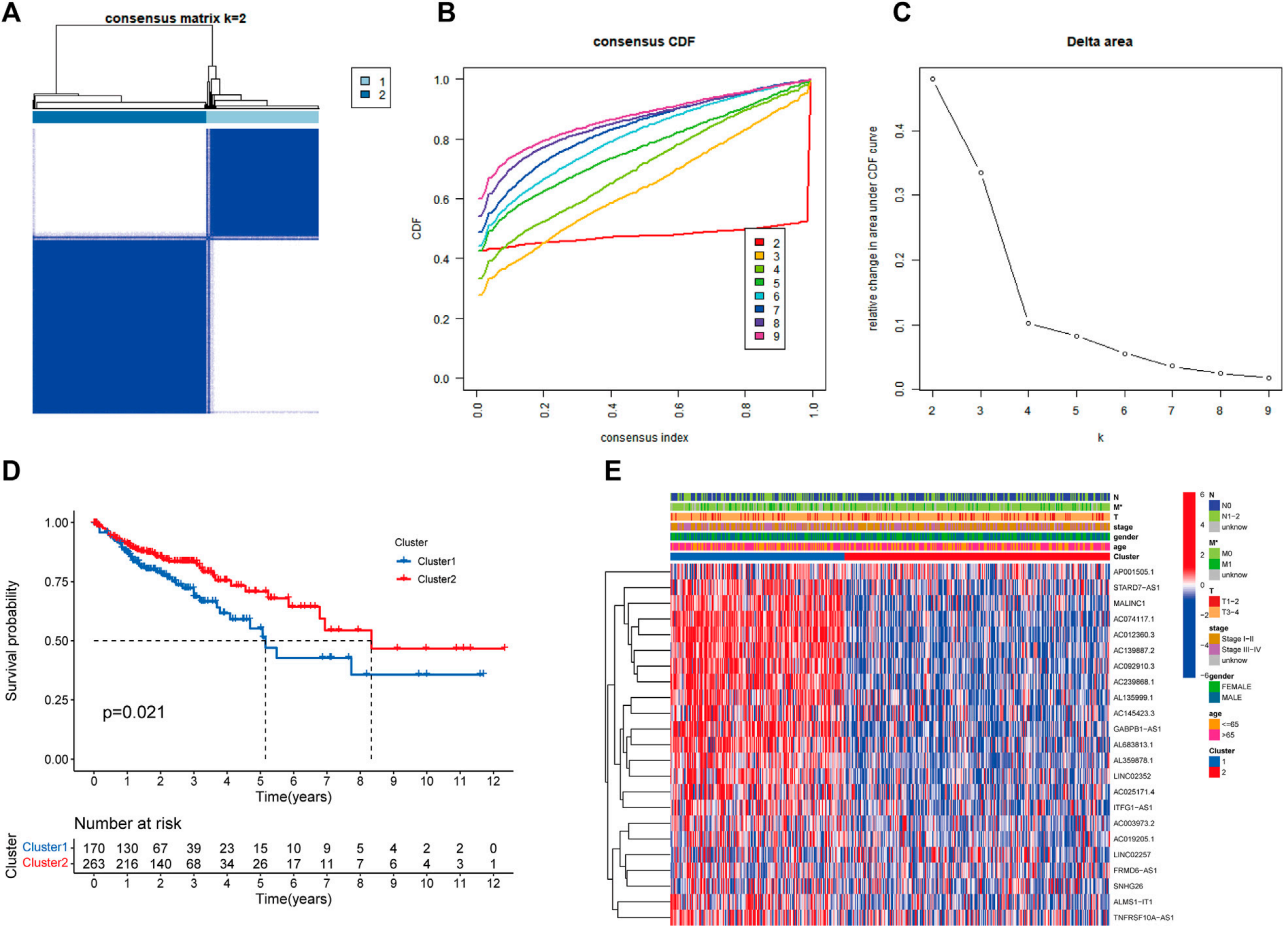
Overall survival and clinical characteristics of different subgroups of CC. **(A)** Consensus matrix at optimal *k* = 2. **(B)** The cumulative distribution function (CDF) from *k* = 2 to 9. **(C)** Relative variation of the area under the CDF region at *k* = 2–9. **(D)** Kaplan-Meier curves of the overall survival (OS) time of cluster 1 and cluster 2 (*p* = 0.021). **(E)** Heatmap of clinical characteristics and 23 prognostic lncRNA expressions among the two clusters. *p<0.05.

Based on the expression levels of 23 prognosis-related RMlncRNAs in CC samples, we clustered 433 samples by an unsupervised clustering approach to further elucidate the biological differences between subgroups. Our results showed that K = 2 was the optimal number of clusters with the highest correlation within groups and the least interference between groups (Figures 4A–C). Therefore, CC patients were divided into two subgroups: Cluster1 (*n* = 170) and Cluster2 (*n* = 263). The survival analysis results showed a significant survival advantage for Cluster2 patients (*p* = 0.021, Figure 4D). The heatmap showed differences in prognosis-related RMlncRNA expression between subgroups (Figure 4E), and most RMlncRNAs were highly expressed in Cluster1. In addition, we found that patients with distant metastasis (M1) were more represented in Cluster1 (*p* < 0.05), while other clinicopathological features were not significantly different between the two subgroups.

### Characterization of Immune Microenvironmental Infiltration Between the Distinct Clusters

**FIGURE 5:**
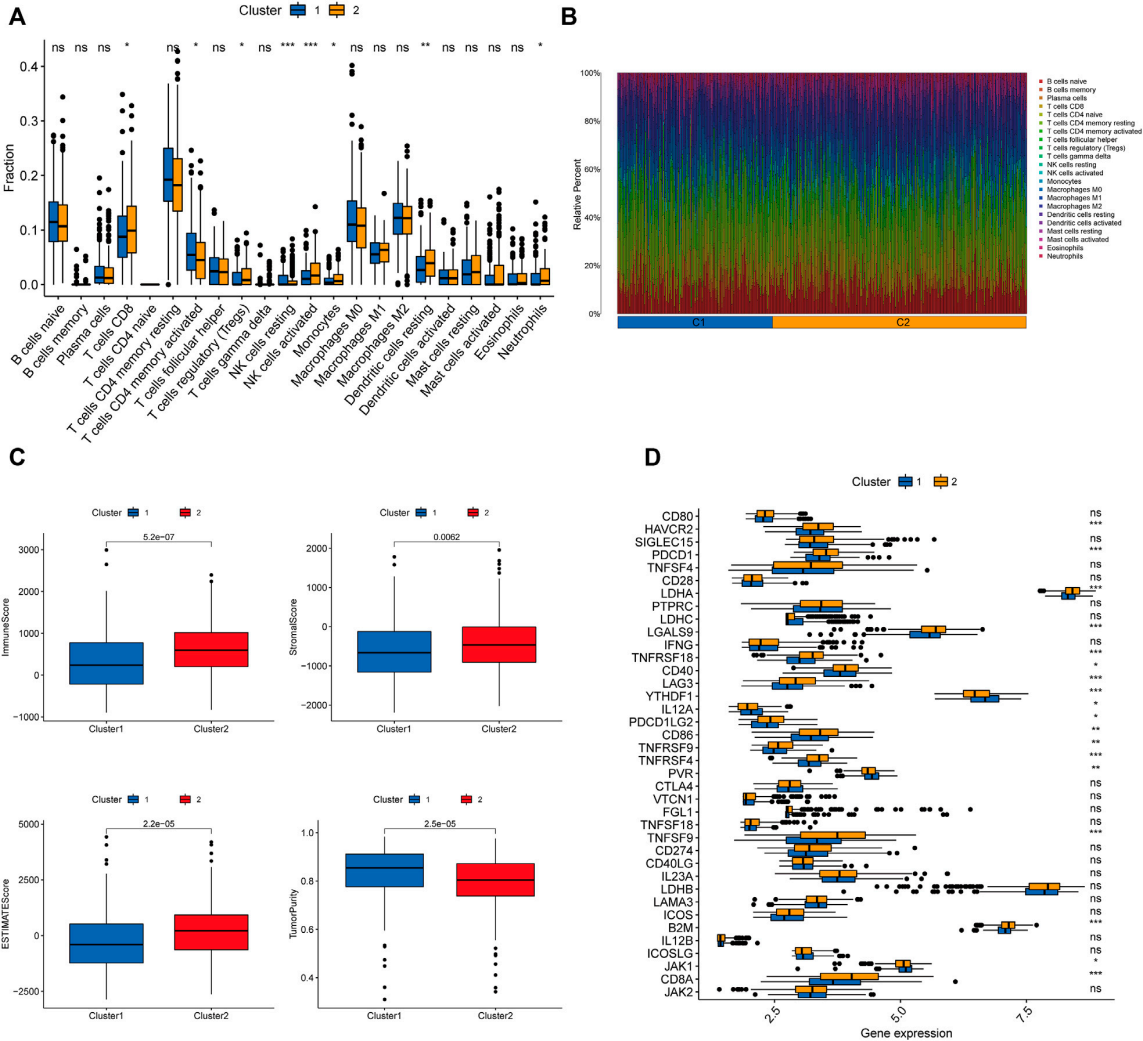
Characterization of TME cell infiltration in different clusters. **(A)** CIBERSORT analysis of the abundance of 22 tumor-infiltrating immune cells (TICs) infiltration between the two groups. **(B)** The bar graph displaying the ratio of 22 TICs types for CC patients in cluster 1 and cluster 2. **(C)** The violin plots depicting the difference in tumor microenvironment scores (including immune score, stromal score, ESTIMATE score, and tumor purity) between the two clusters. **(D)** Expression of 32 immune checkpoint genes between the two clusters. ∗*p* < 0.05; ∗∗*p* < 0.01; ∗∗∗*p* < 0.001;ns, no sense.

We further explored the differences in immune microenvironment characteristics between distinct clusters to understand the interactions between RNA methylation-related lncRNAs and the immune microenvironment (TME). The results of CIBERSORT analysis (Figure 5A) showed that 8 of the 22 immune infiltrating cells differed between clusters, with T cells CD8, T cells regulatory (Tregs), NK cells resting, NK cells activated, monocytes, dendritic cells resting, and neutrophils showed more infiltration in Cluster2, while only T cells CD4 memory activated were highly enriched in Cluster1. The percentages of 22 immune cell types in GC patients between the two clusters are shown in Figure 5B. ESTIMATE analysis showed (Figure 5C) that the immune score (*p* < 0.001), stromal score (*p* = 0.0062), and ESTIMATE score (*p* < 0.001) were significantly higher in Cluster2 than Cluster1, while the tumor purity in Cluster1 (*p* < 0.001) was considerably higher than Cluster2. In addition, we tried to determine the correlation between subgroups and some immune checkpoints. We found remarkable differences in the expression levels of 18 immune checkpoint genes between the two subtypes (*p* < 0.05). The expression levels of PD-1, PD-L1, HAVCR2, CTLA4, LDHA, LGALS9, TNFRSF18, YTHDF1, LAG3, CD40, TNFRSF4, TNFRSF9, CD86, B2M, and CD8A were higher in Cluster2, whereas PDCD1LG2, IL12A, PVR, and JAK1 were higher in Cluster 2 (Figure 5D). Previous studies have shown that high immune scores and activation of suppressive immune checkpoints (like HAVCR2, PD-L1, CTLA-4) play a crucial role in “hot tumors” (Zhan et al., 2021). “Hot tumors” are more likely to benefit from immune checkpoint blockade (ICB) therapy, whereas “cold tumors” with low levels of immune infiltration are more likely to become resistant to immunotherapy (Galon and Bruni, 2019). Therefore, we may consider cluster 1 as the “cold tumor” and cluster 2 as the “hot tumor”, which may predict different immunotherapy responses.

### Construction and Validation of RNA Methylation-Related lncRNA Prognostic Signature

**FIGURE 6:**
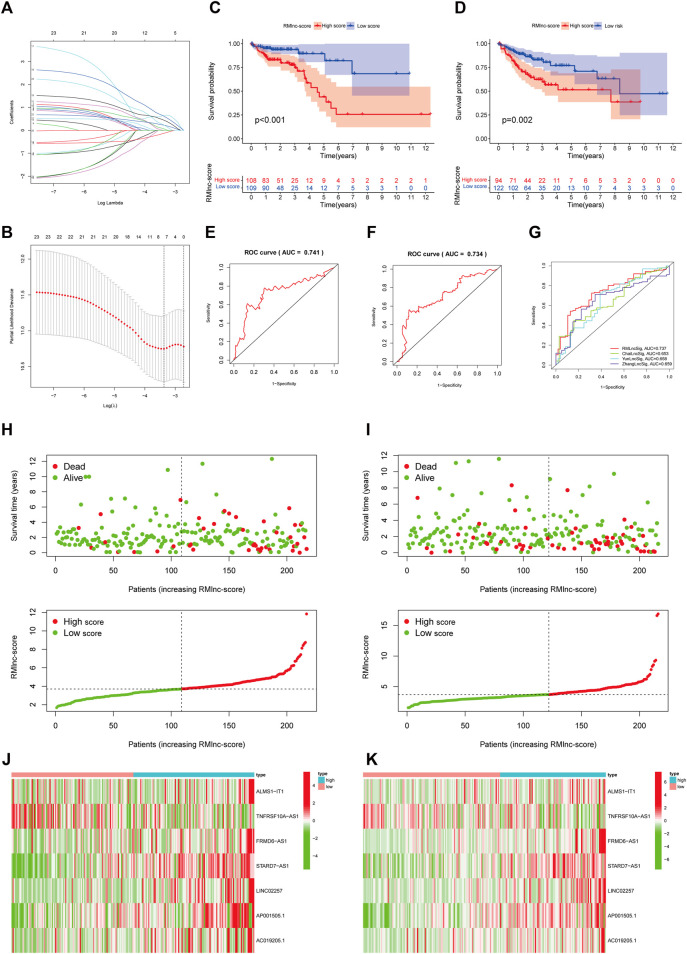
RNA methylation-related lncRNA prognostic signature. **(A,B)** Seven optimal RNA methylation-related lncRNAs were found using the least absolute shrinkage and selection operator (LASSO) cox regression. **(C,D)** Kaplan-Meier curves for overall survival in the training and test sets. **(E,F)** ROC curves were used to predict the 5-years survival of patients in the training and test sets. The AUC was 0.741 in the training set and 0.734 in the test set. **(G)** Comparison of RMlinc-score with other prognostic evaluation models. **(H,I)** Survival status and RMlinc-score curves in the training and test sets. **(J,K)** Heatmap of RNA methylation-related lncRNAs expression in the training and test sets.

**FIGURE 7:**
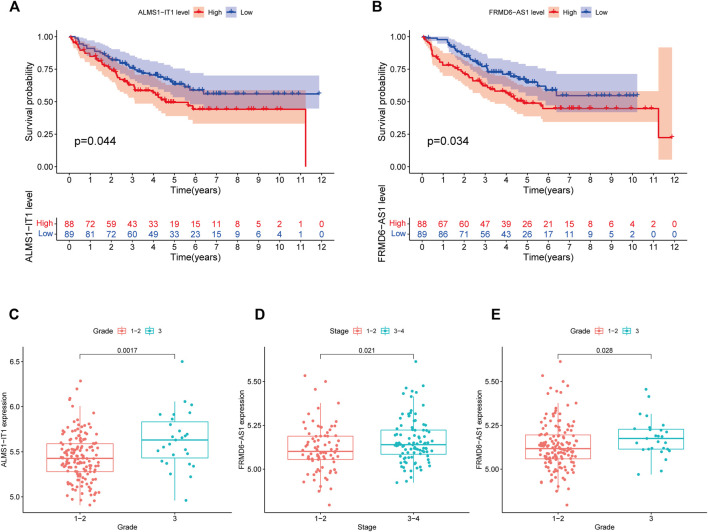
Validation of lncRNA prognostic signatures in the GEO cohort. **(A)** Kaplan-Meier survival curve of ALMS1-IT1. **(B)** Kaplan-Meier survival curves of FRMD6-AS1. **(C)** Correlation between ALMS1-IT1 expression and grade. **(D)** Correlation between FRMD6-AS1 expression and stage. **(E)** Correlation between FRMD6-AS1 expression and grade.

The 433 colon cancer patients were randomly divided into a training set (*n* = 217) and a test set (*n* = 216). To avoid overfitting, we screened the seven most powerful prognostic RMlncRNAs by LASSO regression analysis, which were used to construct the RNA methylation-related lncRNA prognostic signature (RMlnc-score) (Figures 6A, B). The correlation coefficients are shown in Table 1. Patients were classified into low RMlnc-score and high RMlnc-score groups according to the cut-off values of RMlnc-score. The RMlnc-score for each patient was calculated as follows:RMlnc-score=(0.0645*ALMS1-IT1 expression) + (−0.1268*TNFRSF10A-AS1 expression) + (0.6464*FRMD6-AS1 expression) + (0.6173*STARD7-AS1) + (0.4430*LINC02257 expression) + (0.2254*AP001505.1 expression) + (0.2329*AC019205.1 expression). The Kaplan-Meier curves showed that in the training set (*p* < 0.001, Figure 6C) and test set (*p* = 0.002, Figure 6D), patients in the high RMlnc-score group had a worse prognosis compared to the low RMlnc-score group. The area under the curve (AUC) for 5-years overall survival (OS) was 0.741 and 0.734 for the training and test sets, respectively (Figures 6E, F). In the overall cohort (Figure 6G), the RMlnc-score (our study) had an AUC of 0.737 at 5-years overall survival, which was substantially higher than ChaiLncSig (AUC = 0.653), YunLncSig (AUC = 0.658), and ZhangLncSig (AUC = 0.659). This suggests that the RMlnc-score has higher accuracy in predicting survival compared to three recently published lncRNA signatures for colon cancer (Chai et al., 2021; Yun and Yang, 2021; Zhang et al., 2021d). The survival status and RMlnc-score score curves for the training and test sets showed (Figure 6H, I, 7I) that RMlnc-score was proportional to the number of deaths in CC patients. The heatmaps showed (Figures 6J, K) that the expression of ALMS1-IT1, FRMD6-AS1, STARD7-AS1, LINC02257, AP001505.1, and AC019205.1 was upregulated in the high RMlnc-score group, while TNFRSF10A-AS1 was upregulated in the low RMlnc -score group was up-regulated. In addition, we performed a validation analysis of the signature lncRNA in the GSE17536 cohort. However, due to fewer non-coding genes in the microarray data, we only detected ALMS1-IT1 and FRMD6-AS1. Our results showed that high expression of ALMS1-IT1 (*p* = 0.044) and FRMD6-AS1 (*p* = 0.034) was significantly associated with poor prognosis of patients (Figures 7A, B). High expression of ALMS1-IT1 was associated with high grade (*p* = 0.0017, Figure 7C) and high expression of FRMD6-AS1 was associated with high stage (*p* = 0.021, Figure 7D) and high grade (*p* = 0.028, Figure 7E).

**TABLE 1:** The correlation coefficients of 7 RNA methylation-related lncRNAs.

Gene	Coef
ALMS1-IT1	0.064532959
TNFRSF10A-AS1	−0.12683763
FRMD6-AS1	0.64643134
STARD7-AS1	0.617319233
LINC02257	0.443023664
AP001505.1	0.225414755
AC019205.1	0.232908459

### Independent Prognostic and Clinical Correlation Analysis

**FIGURE 8:**
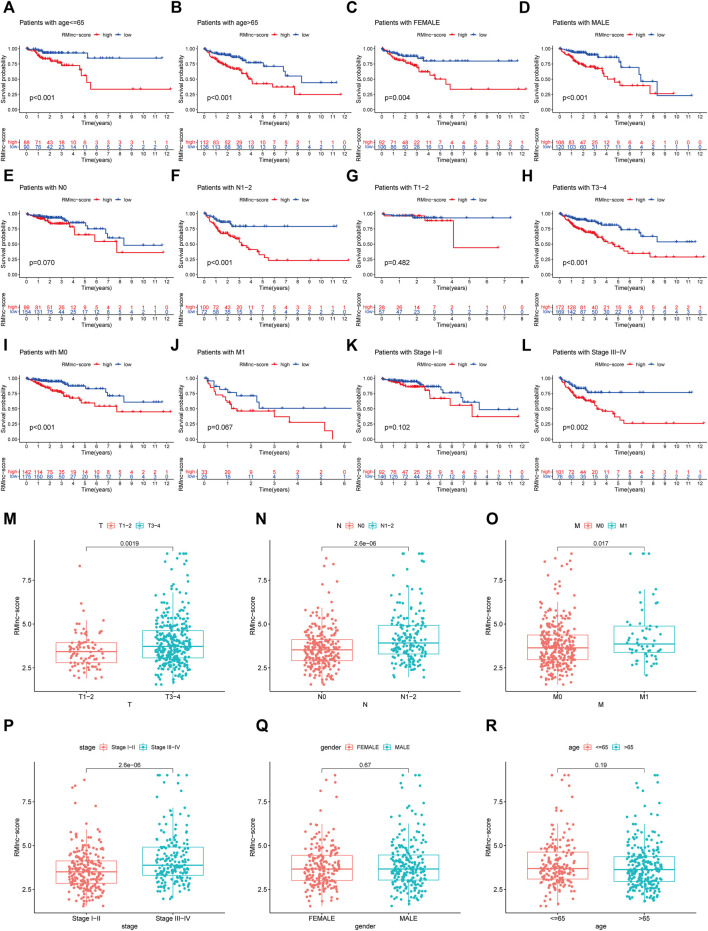
Correlation of clinical characteristics with RMlnc-score by subgroup analysis. Kaplan-Meier curves stratified by **(A,B)** age, **(C,D)** sex, **(E,F)** N stage, **(G,H)** T stage, **(I,J)** M stage, and **(K,L)** clinical stage. **(M–R)** Differential analysis of RMlnc-score for different subgroups.

**FIGURE 9:**
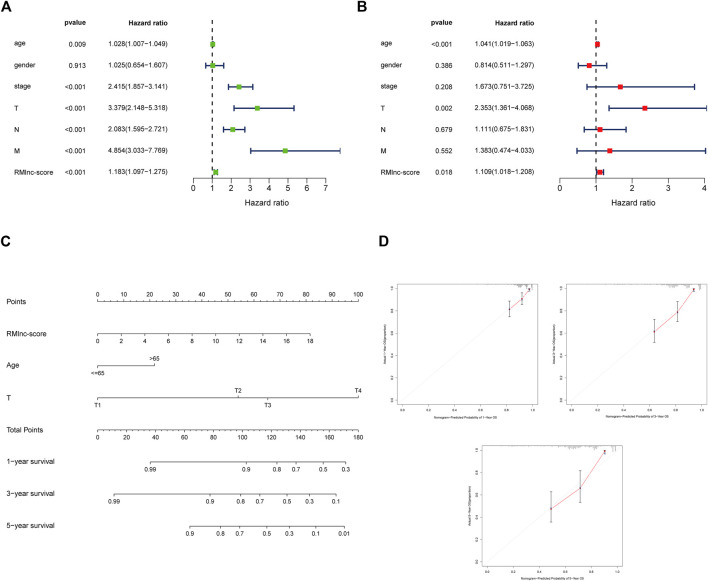
Establishment of nomogram for predicting OS in colon cancer patients. **(A)** Univariate Cox regression analysis of clinical characteristics and RMlnc-score in CC samples. **(B)** Multivariate Cox regression analysis of clinical characteristics and RMlnc-score in CC samples. **(C)** The nomogram with multiple independent predictors, including age, T-stage, and RMlnc-score, was employed to predict 1-, 3-, and 5-years OS in patients with colon cancer. **(D)** Calibration curves of the nomogram for predicting 1,3,5-years OS.

Stratified survival analysis in combination with clinical characteristics (Figures 8A–L) showed that in age>65 (*p* < 0.001), age ≤ 65 (*p* < 0.001), male (*p* < 0.001), female (*p* = 0.004), stage III-IV (*p* = 0.002), T3-4 (*p* < 0.001), M0 (*p* < 0.001), and N1-2 (*p* < 0.001) subgroups of patients, survival was significantly lower in the high RMlnc-score group than in the low RMlnc-score group. By comparing the RMlnc-score of patients in different groups, we found that RMlnc-score increased with increasing T-stage, N-stage, M-stage, and clinical stage, while no significant differences were seen for age and gender (Figures 8M–R). Univariate Cox regression analysis showed that age, stage, T-stage, N-stage, M-stage, and RMlnc-score (all *p* < 0.001) were strongly associated with prognosis (Figure 9A). Multivariate Cox regression analysis confirmed that age, T-stage, and RMlnc-score were independent prognostic factors for CC patients (Figure 9B). Based on the three independent prognostic factors in the multivariate Cox regression analysis, we created a nomogram capable of predicting the incidence of OS in CC patients at 1, 3, and 5 years (Figure 9C). The calibration curve demonstrated the high accuracy and sensitivity of this nomogram (Figure 9D).

### PCA Analysis and Immune Microenvironment Characterization

**FIGURE 10:**
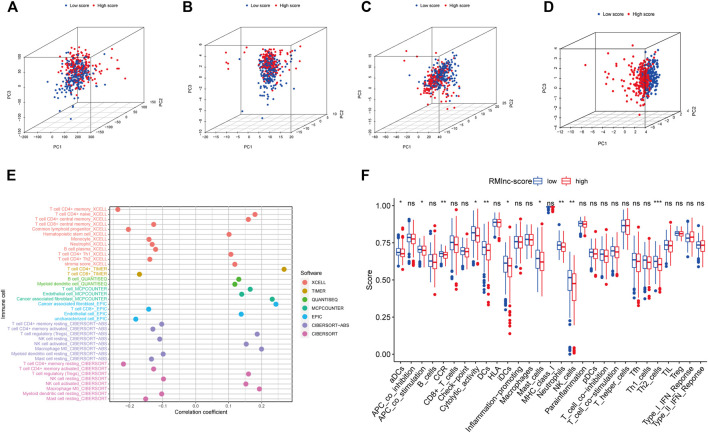
The principal component analysis and immune microenvironment differences of high and low RMlnc-score groups. Principal component analysis between low RMlnc-score and high RMlnc-score groups based on the expression of **(A)** all genes, **(B)** RNA methylation-related genes, and **(C)** RNA methylation-related lncRNAs and the **(D)** seven lncRNAs of prognostic signature. **(E)** Correlation between RMlnc-score and tumor-infiltrating immune cells. The correlation coefficient higher than 0 indicated positive correlation and lower than 0 denoted negative correlation. **(F)** Differences in immune cells and immune function between the high RMlnc-score and low RMlnc-score groups. *p<0.05; **p<0.01; ***p<0.001; ns, no sense.

The results of principal component analysis (PCA) showed no significant differences between the high RMlnc-score group and the low RMlnc-score group in the expression of all genes (Figure 10A), RNA methylation-related genes (Figure 10B), and RNA methylation-related lncRNAs (Figure 10C). However, in the expression of the seven lncRNAs used in the prognostic model (Figure 10D), there was a significant difference between the high RMlnc-score and low RMlnc-score groups. We also explored whether our model could predict immune cell infiltration in CC. The bubble plot (Figure 10E) showed that RMlnc-score was positively correlated with CD4^+^ T cells, cancer-associated fibroblast (CAFs), myeloid dendritic cell, macrophage M0, NK cell activated, hematopoietic stem cell while negative correlation with CD4+8 cell, monocyte, neutrophil, and B cell plasma. The ssGSEA results (Figure 10F) showed that some immune cells, including dendritic cells (DCs), activated dendritic cells (aDCs), immature dendritic cells (iDCs), mast cells, neutrophils, NK cells, and type 2 T helper were significantly increased in the low RMlnc-score group, and some pathways associated with immune function, namely APC co-stimulation, C-C chemokine receptor, and cytolytic activity, were significantly activated in the low RMlnc-score group.

### Immunotherapy Response Analysis

**FIGURE 11:**
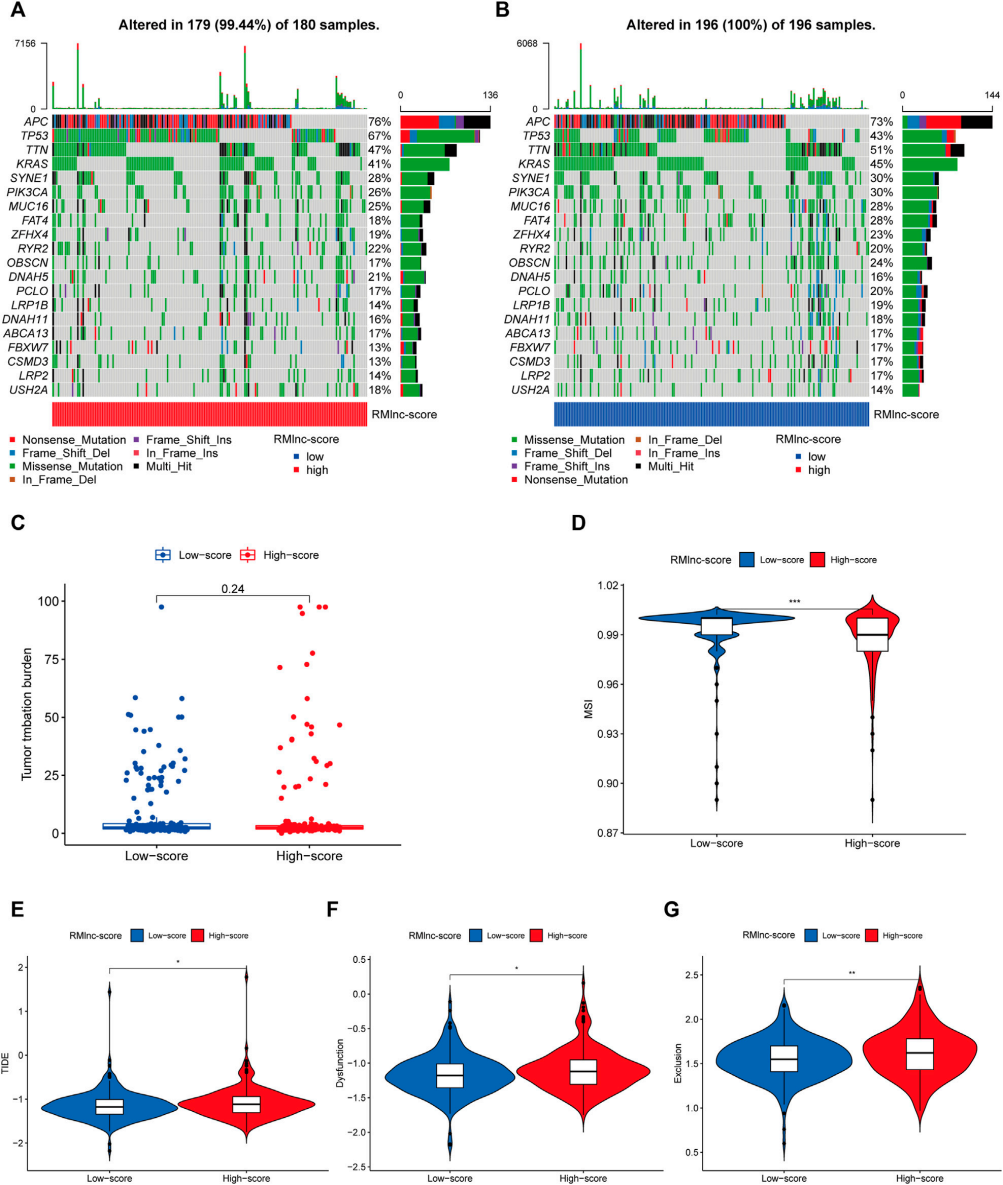
Predictability of immunotherapy response in the prognostic signature. **(A,B)** Waterfall plot of the tumor mutational burden (TMB) landscape in the high RMlnc-score and low RMlnc-score groups presenting the top 20 genes with the highest mutation frequency. **(C)** Differences in TMB of colon cancer patients in the high and low RMlnc-score groups. **(D)** Differences in microsatellite instability (MSI) of colon cancer patients in high and low RMlnc-score groups. **(E-G)** TIDE prediction scores (including TIDE score, dysfunction score, and exclusion score) between high RMlnc-score and low RMlnc-score groups. *p<0.05; **p<0.01.

TMB and MSI have been reported to be predictive biomarkers of immunotherapeutic response (Ock et al., 2017; Vandekerkhove et al., 2021). Therefore, we first compared somatic mutations in high RMlnc-score and low RMlnc-score and visualized the top 20 genes with the highest mutation frequency (Figures 11A, B). However, there was no significant difference in tumor mutational load between the high RMlnc-score and low RMlnc-score groups (Figure 11C). We then compared the differences in MSI distribution between the different scoring groups and found that the low RMlnc-score group was associated with higher microsatellite instability (MSI) (Figure 11D). TIDE, a novel predictive marker of immunotherapy, was better than known immunotherapy biomarkers (including TMB and PD-L1 expression) for response to immunotherapy in certain tumors (Wang et al., 2019). Higher TIDE scores indicate that tumor cells are more likely to induce immune escape, thus indicating a lower response rate to immunotherapy. Surprisingly, we found that patients in the low RMlnc-score group had significantly lower TIDE scores (including T cell dysfunction and exclusion scores) than those in the high RMlnc-score group (Figures 11E–G). The above findings suggested that RMlnc-score correlates with the response of CC patients to immunotherapy and may help predict the efficacy of ICB immunotherapy.

### Drug Sensitivity Analysis

**FIGURE 12:**
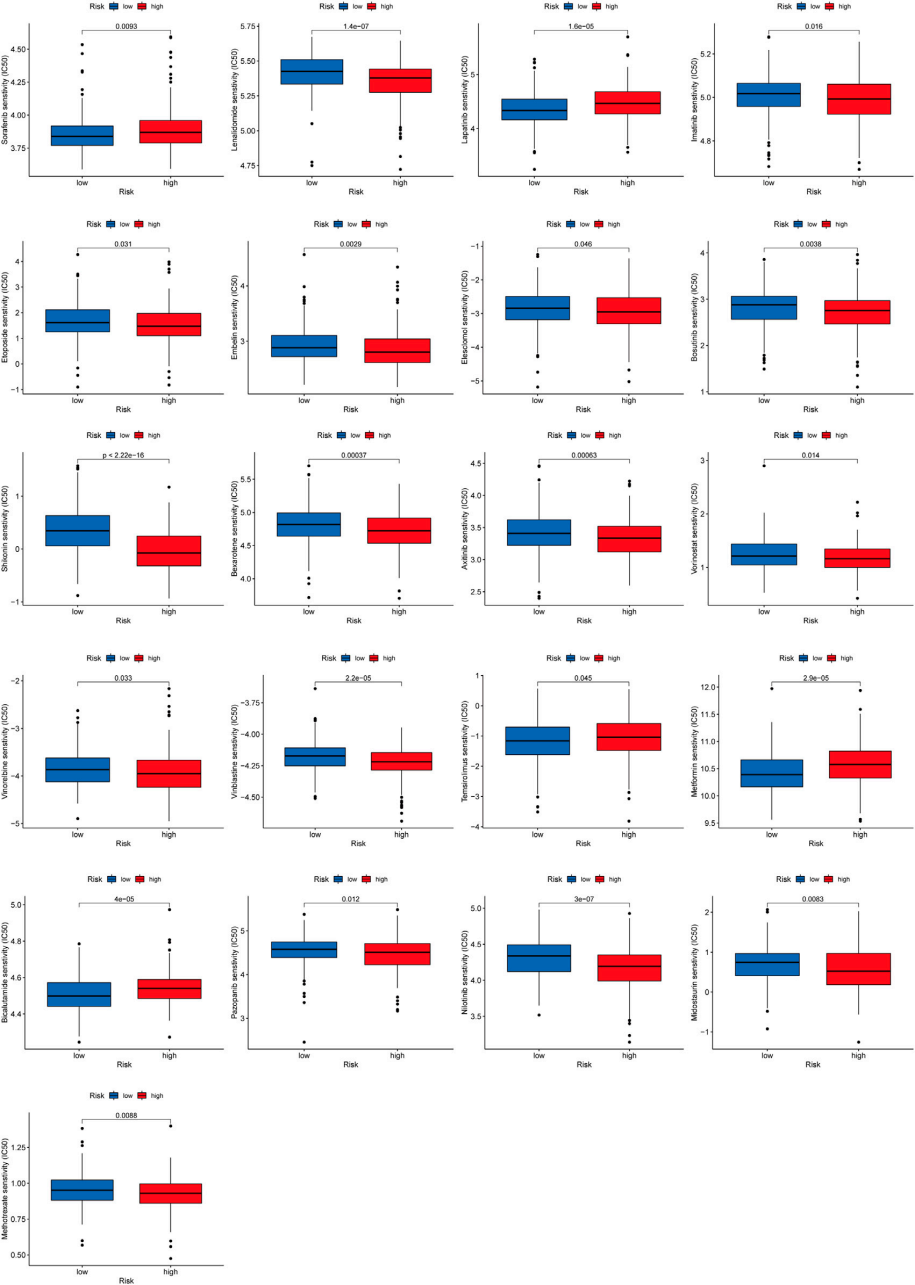
Anti-tumor drug sensitivity in high RMlnc-score and low RMlnc-score populations.

To explore the effect of RMlnc-score on drug response, we compared the half-maximal inhibitory concentration (IC50) of the commonly used drugs in both groups. The results showed that the IC50 values of bicalutamide, lapatinib, sorafenib, metformin, and temsirolimus were higher in the high RMlnc-score group, indicating that patients in the low-scoring group were more sensitive to these five drugs. In contrast, axitinib, bexarotene, bosutinib, elesclomol, embelin, etoposide, imatinib, lenalidomide, methotrexate, midostaurin, nilotinib, pazopanib, shikonin, vinblastine, vinorelbine, and vorinostat had higher IC50 in patients with low RMlnc-score, implying that patients in the high RMlnc-score group were more sensitive to these drugs (Figure 12).

### Analysis of RNA Methylation Modification Sites

After scanning the m6A-Atlas, m5C-Atlas, and m7GHub databases, we eventually obtained six m6A, nine m5C, and one m7G modification sites on STARD7-AS1 and five m5C modification sites on FRMD6-AS1, which have been experimentally validated (Supplementary Table S1). Then, we also utilized the widely used bioinformatics tools SRAMP, RNAm5Cfinder, and iRNA-m7G to predict potential m6A, m5C, and m7G modification sites on our seven lncRNAs. Some meaningful results showed that all seven lncRNAs were potentially methylated (Supplementary Table S2).

## Discussion

CC is a highly complex and heterogeneous tumor characterized by high morbidity and poor prognosis (Kumar et al., 2021). Chemotherapy for CC has progressed in recent years, but tumor resistance is frequent when traditional histological and anatomical classifications are used to guide anti-tumor therapy. Therefore, accurate identification of molecular subtypes of CC is vital to guide individualized treatment. Although previous studies have also identified several prognostic signatures of CC for the stratification of colon cancer patients, considerable heterogeneity remains between subtypes (Cui et al., 2021; Song et al., 2021b). Therefore, more accurate prognostic signatures of CC are urgently needed to improve patient survival. An increasing number of studies have shown that RNA methylation modifications (including m6A, m5C, m1A, and m7G) play an essential role in tumor progression and influence specific biological processes by interacting with lncRNAs (Chen et al., 2021; Yao et al., 2021). Huang et al. constructed an m5C-associated lncRNA prognostic signature that accurately predicted breast cancer patient’s prognosis and immune microenvironment characteristics (Huang et al., 2021). A recent study has identified the critical role of m6A/m5C/m1A-related lncRNA-based prognostic signature in predicting molecular subtypes and prognosis of head and neck tumors (Wang et al., 2021a). However, to the best of our knowledge, no prognostic signature based on m6A/m1A/m5C/m7G-related lncRNAs has been found to be accurate and applicable to CC patients.

In this study, we first identified 1057 RNA methylation-associated lncRNAs in the TCGA-CORD cohort, 23 of which were confirmed with prognostic value. In addition, we defined two clusters by consensus clustering analysis to investigate potential molecular subtypes of CC. The results showed that the subtypes were strongly correlated with tumor stage and OS, with cluster 2 having better OS and less distant metastasis than cluster 1, reflecting the association between RNA methylation-associated lncRNAs and CC progression and prognosis. Recent studies have shown that RNA methylation and lncRNAs play a critical regulatory role in the immune system, especially in immune cell infiltration and anti-tumor immune responses (Li et al., 2017; Wu et al., 2020; Eptaminitaki et al., 2021). Based on these findings, we obtained TME scores and immune microenvironmental landscapes for each CC sample to investigate the relationship between clusters, TME, and immune checkpoints. The results showed that TME scores, immune infiltrating cells, and immune checkpoints differed significantly between the two clusters. Among them, cluster 2 had a significantly higher immune score, stromal score, and ESTIMATE score than cluster 1, while cluster 1 had a higher tumor purity than cluster 2. The majority of immune infiltrating cells were enriched in cluster 2, including T cells CD8, Tregs, NK cells resting, NK cells activated, Monocytes Dendritic cells resting, and Neutrophils. In addition, we found that 15 out of 18 differentially expressed immune checkpoint molecules (including PD-1, PD-L1, HAVCR2, CTLA4, LDHA, LGALS9, TNFRSF18, YTHDF1, LAG3, CD40, TNFRSF4, TNFRSF9, CD86, B2M, and CD8A) were highly expressed in cluster 2. It was reported that high PD-L1 expression/infiltrating tumors with high immune scores are usually considered hot tumors which are sensitive to immunotherapy. In contrast, low PD-L1 expression/non-infiltrating tumors with low immune scores are typically regarded as cold tumors which are less effective for immunotherapy (Kuriyama et al., 2020). Therefore, we identified cluster 2 as “hot tumor” and cluster 1 as “cold tumor,” corresponding to different prognoses and immunotherapeutic responses.

Among the 23 RNA methylation-related lncRNAs, seven lncRNAs were used to generate prognostic gene signatures that stratified CC patients into low RMlnc-score and high RMlnc-score groups with different OS. The survival time of patients in the high RMlnc-score group was significantly shorter than that in the low RMlnc-score group, both in the training and test sets, which also demonstrated that the prognostic model consisting of all seven lncRNAs could well predict the prognosis of CC patients. We validated the predictive ability of RMlnc-score in patients stratified based on clinicopathological parameters. We noticed that RMlnc-score showed a strong positive correlation with tumor progression (T3-4, N1-2, M1, and stage III-IV). Univariate and multivariate cox regression analyses showed that RMlnc-score, age, and T-stage were available as independent prognostic factors for OS in CC patients. By integrating these independent prognostic factors, we constructed nomograms that could predict 1-, 3-, and 5-years survival in CC patients, which were highly accurate and reliable in estimating individual survival rates. Notably, we further validated the correlation of our signature lncRNA with clinicopathological features in the GSE17536 cohort. We detected that high expression of lncRNAs ALMS1-IT1 and FRMD6-AS1 was associated with poorer prognosis and poorer differentiation. High FRMD6-AS1 expression was also associated with higher clinical stage. Previous studies have shown that upregulation of ALMS1-IT1 can promote lung cancer progression by mediating AVL9 activation of the cell cycle protein-dependent kinase pathway (Luan et al., 2021). Li et al. constructed a ferroptosis-related lncRNA prognostic signature that also included ALMS1-IT1 and found it to be strongly associated with poor prognosis in colon cancer (Li et al., 2022). These findings validated the oncogenic properties of ALMS1-IT1 and are consistent with our results. Unfortunately, there are few studies on the remaining lncRNAs. Therefore, we anticipated that our results would help to demonstrate the prognostic value of these RNA methylation-related lncRNAs, thus providing insights into their potential role in carcinogenesis and progression of CC.

Currently, only a minority of CC patients have responded to immunotherapy in clinical practice. Thus, it is necessary to assess the value of prognostic characteristics in predicting response to immunotherapy. The effectiveness of immunotherapy is influenced by the immunogenicity of the tumor microenvironment, which is why understanding TME is essential for evaluating immunotherapy (Turley et al., 2015). The ssGSEA results showed that the low RMlnc-score group had a greater enrichment of immune-related cells and immune-related pathways, including dendritic cells (DCs), activated dendritic cells (aDCs), immature dendritic cells (iDCs), mast cells, neutrophils, NK cells, type 2 T helper, APC co-stimulation, C-C chemokine receptor, and cytolytic activity. The above results indicated that patients with low RMlnc-score had higher immunogenicity and better immunotherapy response.

Drug efficacy is related to drug sensitivity and individual differences in patients, and targeting the appropriate subpopulation will improve drug efficacy. Therefore, we further analyzed the sensitivity of patients in distinct RMlnc-score groups to anti-tumor drugs. Prediction of chemotherapy drug sensitivity showed that bicalutamide, lapatinib, sorafenib, metformin, and temsirolimus were the ideal choices for CC patients in the low RMlnc-score group. At the same time, axitinib, bexarotene, bosutinib, elesclomol,embelin, etoposide, imatinib, lenalidomide, methotrexate, midostaurin, nilotinib, pazopanib, shikonin, vinblastine, vinorelbine, and vorinostat may work better in patients in the high RMlnc-score group.

However, our study has some limitations. First, this is a retrospective analysis based on an online public database, and we used internal validation methods in the TCGA cohort and external validation in the GSE17536 independent cohort, but large-scale prospective data are still needed to validate our prognostic signature. In addition, the potential mechanism of RMlnc-score may need further validation by *in vitro* and *in vivo* experiments.

## Conclusion

In summary, our study elucidated that RNA methylation-related lncRNAs and can predict the prognosis of CC patients and guide more effective and personalized treatment strategies by identifying hot and cold tumors. Targeting RNA methylation and lncRNAs would be a promising way to overcome individual treatment failure and improve patient prognosis.

## Data Availability

The original contributions presented in the study are included in the article/Supplementary Material, further inquiries can be directed to the corresponding author/s.
